# Characteristics of surgically resected non‐small cell lung cancer patients with post‐recurrence cure

**DOI:** 10.1111/1759-7714.13669

**Published:** 2020-09-22

**Authors:** Dai Sonoda, Yosuke Matsuura, Yasuto Kondo, Junji Ichinose, Masayuki Nakao, Hironori Ninomiya, Yuichi Ishikawa, Makoto Nishio, Sakae Okumura, Yukitoshi Satoh, Mingyon Mun

**Affiliations:** ^1^ Department of Thoracic Surgical Oncology The Cancer Institute Hospital, Japanese Foundation for Cancer Research Tokyo Japan; ^2^ Department of Thoracic Surgery Kitasato University School of Medicine Kanagawa Japan; ^3^ Department of Pathology The Cancer Institute Hospital, Japanese Foundation for Cancer Research Tokyo Japan; ^4^ Department of Thoracic Medical Oncology The Cancer Institute Hospital, Japanese Foundation for Cancer Research Tokyo Japan

**Keywords:** Lung, radiotherapy, recurrence, thoracic surgery

## Abstract

**Background:**

The prognosis of postoperative recurrence in patients with non‐small cell lung cancer (NSCLC) is poor. However, depending on the recurrence patterns and treatment options, some patients can achieve long‐term survival following recurrence. In this study, we investigated the clinicopathological characteristics of NSCLC patients with curable disease who developed postoperative recurrence.

**Methods:**

This retrospective study enrolled 535 patients who had developed recurrence from among 1760 consecutive patients with NSCLC who underwent curative resection from 1990 to 2008.

Post‐recurrence cure was defined as being cancer‐free for at least five years after treatment for recurrence in patients who had undergone radical local treatment or chemotherapy. The clinicopathological characteristics associated with post‐recurrence cure were analyzed.

**Results:**

Among 535 patients who developed recurrence, 24 (4.5%) achieved post‐recurrence cure. The median post‐recurrence follow‐up duration was 151 (85–275) months for those who achieved post‐recurrence cure. The solitary recurrent lesions and local treatment for the initial recurrence site were significantly more for patients who could be cured after they developed recurrence. All patients with post‐recurrence cure received only radical local treatment for the recurrent lesions.

**Conclusions:**

Some patients with solitary recurrent NSCLC lesions can be cured with only radical local treatment.

**Key points:**

**Significant findings of the study**

The post‐recurrence cure patients maintained a cancer‐free status for five years after treatment for recurrence without a second recurrence. All patients with post‐recurrence cure received only radical local treatment for recurrence and had significantly higher number of solitary recurrent lesions.

**What this study adds**

Some patients with solitary recurrent NSCLC lesions after resection can be cured with only radical local treatment.

## Graphical abstract

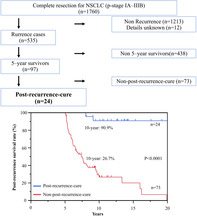

Among 535 patients who developed recurrence, 24 (4.5%) patients achieved post‐recurrence cure. The post‐recurrence cure patients who sustained cancer‐free status for five years after treatment for recurrent lesions did not develop second recurrence. All post‐recurrence cure patients received only local treatment, and the solitary recurrent lesions and local treatment at the initial recurrent sites were significantly associated with post‐recurrence cure.

## Introduction

Complete surgical resection is considered to be the most curative treatment for non‐small cell lung cancer (NSCLC). Although curative resection is performed, about 30%–70% of these patients develop recurrence.^1^, ^2^, ^3^, ^4^ Postoperative recurrence status is considered a systemic disease, that is associated with poor outcomes and a median survival duration of 11.4–17.7 months.^5^, ^6^, ^7^ Therefore, treatment strategies for postoperative recurrence cases are important issues.

With respect to the postoperative recurrence state of NSCLC patients, several recent studies have reported on the importance of radical local treatments for recurrent lesions and have examined factors associated with overall survival and post‐recurrence survival (PRS).^4^, ^8^, ^9^, ^10^, ^11^, ^12^, ^13^ In fact, some patients achieved long‐term PRS (≥5 years), depending on the recurrence patterns and treatment options. However, few studies have investigated the characteristics of these patients.

Therefore, we aimed to determine the clinicopathological features of patients with postoperative recurrence who achieved long‐term PRS and were considered to be cured after post‐recurrence treatment.

## Methods

### Patient selection and data collection

This study performed a retrospective review of patient records of 1760 consecutive patients with pathological stage IA–IIIB NSCLC who underwent curative resection with systematic lymph node dissection from January 1990 to December 2008 at the Cancer Institute Hospital of Japanese Foundation for Cancer Research, Tokyo, Japan. Curative resection was defined as cancer‐negative surgical margins on both macroscopic and histological assessment.

**Figure 1:**
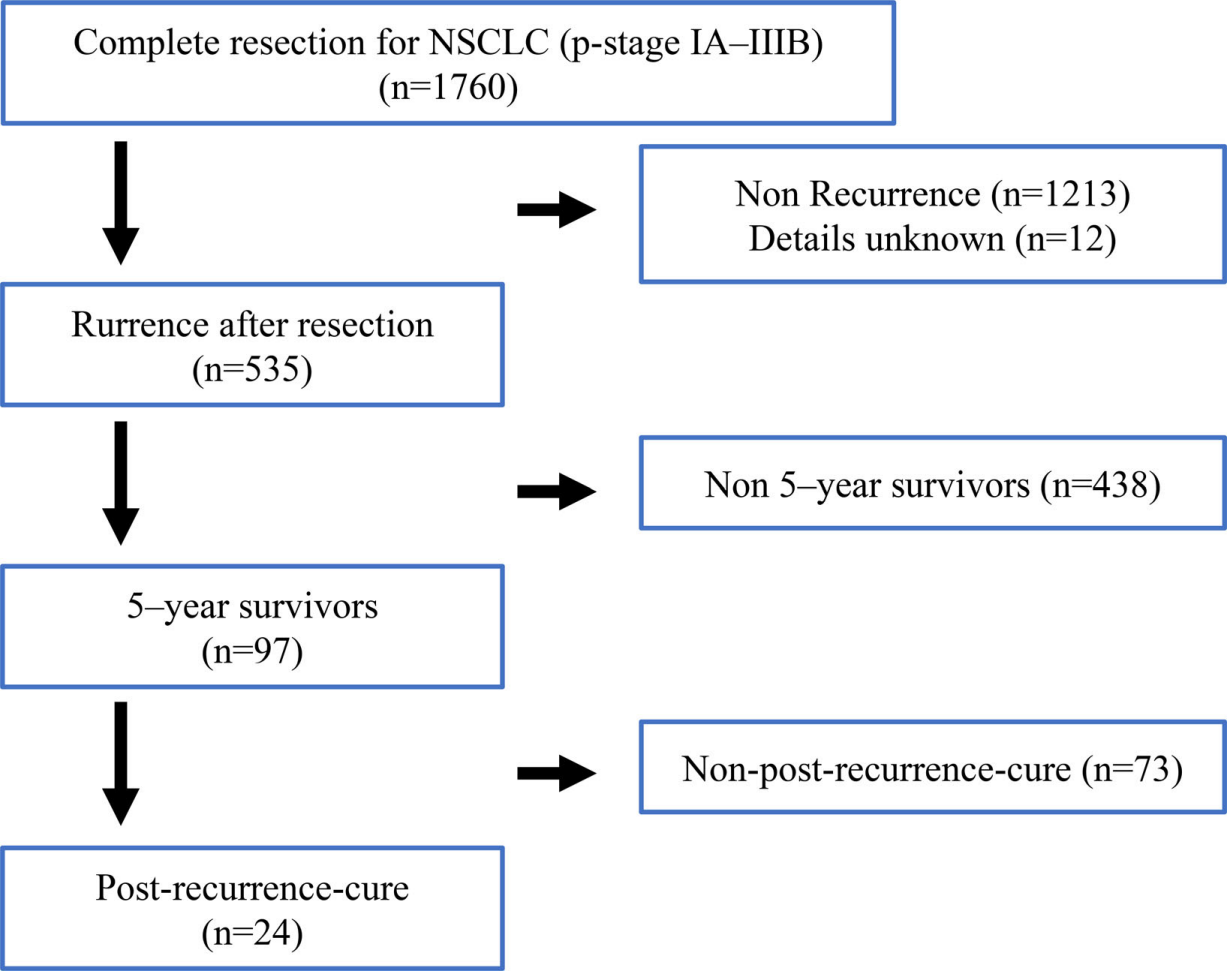
The schematic diagram of the study.

The cohort assessment based on patient medical records revealed that 547 (31.1%) patients had developed postoperative recurrence. After excluding 12 patients for whom detailed recurrence status or information was unavailable, 535 patients were finally enrolled. The characteristics of the enrolled patients are shown in Fig 1.

This study was performed as per the amended Declaration of Helsinki and was approved by the Institutional Review Board of Cancer Institute Hospital of Japanese Foundation for Cancer Research in Tokyo, Japan (approval No. 2019‐1151). The need to obtain written informed consent from each patient was waived because of the retrospective nature of the study and anonymity of the subjects.

Pathological staging was based on the seventh edition of the Union for International Cancer Control for Cancer staging system.^14^ Histological diagnosis was established as per the World Health Organization tumor classification.^15^

The candidate baseline variables of analyses were sex, age at first surgery, age at recurrence, smoking history, histology, initial pathological stage, postoperative adjuvant chemotherapy, recurrence‐free survival (RFS), initial recurrence site, number and location of recurrence, and type of post‐recurrence treatment.

The gene mutation status, including that of *EGFR*, was examined in only 19.6% (105/535) of the patients who were involved in the final analyses; therefore, gene mutation status was not included as a study variable.

### Assessment of the recurrence prognosis in patients

The clinicopathological characteristics of the five‐year survivors after initial recurrence were compared with those of five‐year nonsurvivors. Subsequently, we classified the five‐year survivors into the following two groups: post‐recurrence cure and non‐post‐recurrence cure. We defined post‐recurrence cure as the presence of sustained cancer‐free status for five years after treatment for recurrence in patients undergoing radical local treatment or those receiving chemotherapy. The others were defined as those with non‐post‐recurrence cure. Post‐recurrence cure was based on the evaluation of clinical and imaging findings. We also compared the clinicopathological characteristics of the two groups.

The prognosis of patients who developed recurrence was assessed as per the PRS, and independent prognostic factors for PRS were investigated using a Cox proportional hazard model.

### Follow‐up after resection and recurrence

After resection, all patients received routine follow‐up evaluation at six‐month intervals for five years and annually thereafter until 10 years after resection. The survival status of patients who were lost to follow‐up in outpatient consultation was confirmed via telephone or documentation with the patient or the city office.

Routine follow‐up evaluation included physical examination, chest radiography, and blood examination including tumor markers. Additionally, annual chest computed tomography (CT) scans were performed. Further evaluations, including CT scans of the chest and abdomen, brain magnetic resonance imaging, bone scintigraphy, and positron emission tomography‐CT (PET‐CT), were performed in the presence of symptoms or signs of recurrence. Patients treated for recurrence were followed at three to six month intervals by the approaches utilized during the postoperative follow‐up period.

### Evaluation and treatment of patients with postoperative recurrence

The recurrence diagnosis, based on physical examination and diagnostic imaging, was confirmed with histological and/or cytological evaluation where possible. Recurrences were distinguished from second primary tumors based on the criteria given by Martini and Melamed^16^; the multidisciplinary team comprised thoracic surgeons, medical oncologists, radiologists, and pathologists who conferred and determined whether the tumor was a recurrence or a second primary tumor.

Recurrence was classified into the following two categories: locoregional and systemic. Locoregional recurrence was defined as evidence of tumor within the ipsilateral lung, ipsilateral pleural cavity, mediastinum, and/or bronchi. Systemic recurrence was defined as evidence of tumor in the contralateral lung or outside the hemithorax.

A course of treatment for each recurrent patient was decided by the multidisciplinary team based on a comprehensive decision incorporating performance status, respiratory function, and the patient's social background and preference. The systemic therapy was defined as chemotherapy, including molecular targeted therapy.

The radical local treatment was defined as that performed with curative intent including complete surgical resection, stereotactic ablation radiotherapy (SABR), cerebral stereotactic radiosurgery, other radical radiation therapy of 45 Gy or higher doses, concurrent chemoradiotherapy, proton beam therapy, or cryotherapy.

### Statistical analysis

Categorical data were compared using Pearson's chi‐square test, and continuous data were compared using Student's *t*‐test. Survival rates were estimated using the Kaplan–Meier method, and compared using the log‐rank test. Univariate and multivariate analyses of prognostic factors were performed using the Cox proportional hazards model. *P* < 0.05 was considered statistically significant. PRS was defined as the time from the date of initial recurrence diagnosis to the date of death or the last follow‐up. RFS was calculated from after resection to the date of the first recurrence or last living confirmation.

JMP Pro version 15.0 (SAS Institute, Cary, NC, USA) was used to perform all the statistical analyses.

## Results

### Clinicopathological characteristics of patients who developed recurrence

Clinicopathological characteristics of the patients who developed recurrence are shown in Table 1. Solitary recurrent lesions were recognized in 202 patients (37.8%), and radical local treatment was administered to 315 patients (58.9%) in recurrence patients. The median RFS duration of patients with recurrence was 13 (range: 0–237) months. Three patients had zero RFS after curative resection for primary NSCLC. One patient died because of multiple metastases to the lung, liver, peritoneum, and skin <1 month after recurrence. In the other two patients, multiple recurrent lesions in the lungs and bones were detected.

**Table 1:** Clinicopathological characteristics of five‐year survivors and five‐year nonsurvivors after recurrence

	Total	Five‐year survivors	Five‐year nonsurvivors	
Characteristic	(*n* = 535)	(*n* = 97)	(*n* = 438)	*P*‐value
Age at first surgery, years				
Median (range)	64 (26–86)	62 (26–83)	65 (29–86)	0.009 ^†^
Age at recurrence, years				
Median (range)	66 (28–88)	65 (28–86)	66 (29–88)	0.044 ^†^
Sex				
Male (%)	336 (63%)	51 (53%)	285 (65%)	0.021
Smoking history				
Yes	357 (67%)	50 (52%)	307 (70%)	<0.001
Histology				
Adenocarcinoma	389 (73%)	85 (88%)	304 (69%)	<0.001
Others	146 (27%)	12 (12%)	134 (31%)	
Pathological stage				
I	133 (25%)	34 (35%)	99 (23%)	0.010
I I–III	402 (75%)	63 (65%)	339 (77%)	
Adjuvant chemotherapy (%)	107 (20%)	20 (21%)	87 (20%)	0.866
Recurrence‐free survival, months, median, range				
Initial recurrence site	13 (0–237)	24 (2–213)	12 (0–237)	<0.001 ^†^
Locoregional recurrence	128 (24%)	35 (36%)	93 (21%)	0.002
Systemic recurrence	407 (76%)	62 (64%)	345 (79%)	
Number of initial recurrence sites				
Multiple	333 (62%)	39 (40%)	294 (67%)	<0.0
Solitary	202 (38%)	58 (60%)	144 (33%)	
Breakdown of treatment for initial recurrence				
Radical local treatment	315 (59%)	72 (74%)	243 (56%)	<0.001
Nonradical local treatment	220 (41%)	25 (26%)	195 (45%)	

^†^ Student's *t*‐test.

**Figure 2:**
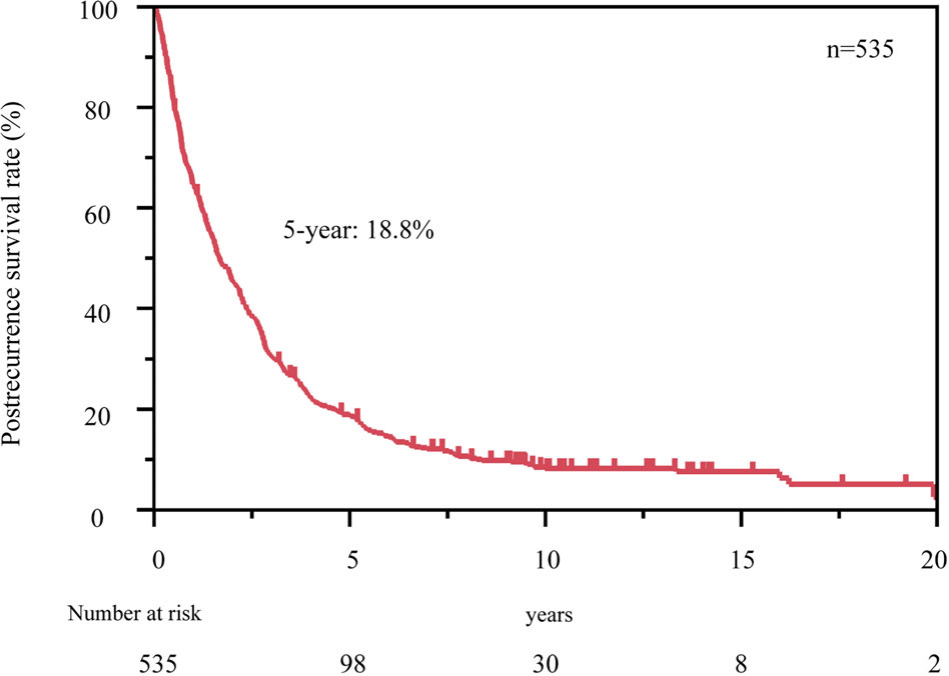
Kaplan–Meier curves for post‐recurrence survival (PRS) in 535 patients with recurrence. The five‐year PRS rate was 18.8%.

The median PRS was 19 (range: 0–275) months, while the five‐year PRS rate was 18.8% (Fig 2). Briefly, 315 patients with recurrence received radical local treatment, including resection alone (51 [16.2%]), resection with radiation therapy (15 [4.8%]), radiation therapy alone (245 [77.8%]), and others (4 [1.3%]). On the other hand, 120 patients received only chemotherapy, and 100 patients received best supportive care.

The prognostic factors associated with PRS are shown in Table 2. Multivariate analysis showed that patients with solitary recurrence and radical local treatment for initial recurrent lesions had significantly improved PRS.

**Table 2:** The prognostic factors associated with PRS in patients with NSCLC recurrence after curative resection

	Univariate		Multivariate	
Characteristics	HR (95% CI)	*P*‐value^†^	HR (95% CI)	*P*‐value^†^
Age at recurrence (years)				
<60	1	0.426	1	0.387
≥60	1.08 (0.89–1.32)		1.09 (0.89–1.34)	
Sex				
Female	1	<0.001	1	0.877
Male	1.44 (1.20–1.73)		0.98 (0.77–1.25)	
Smoking history				
No	1	<0.001	1	<0.001
Yes	1.72 (1.42–2.08)		1.70 (1.32–2.19)	
Histology				
Adenocarcinoma	1	<0.001	1	<0.001
Others	1.97 (0.05–1.00)		1.74 (1.41–2.14)	
Pathological stage				
I	1	0.001	1	0.040
II and III	1.41 (1.14–1.74)		1.26 (1.01–1.57)	
Adjuvant chemotherapy at initial recurrence				
Yes	1	0.195	1	0.118
No	1.16 (0.93–1.45)		1.21 (0.95–1.53)	
Recurrence‐free survival (year)				
>1	1	<0.001	1	<0.001
≤1	1.92 (1.60–2.30)		1.70 (1.41–2.06)	
Initial recurrence site				
Locoregional recurrence	1	<0.001	1	0.196
Systemic recurrence	1.51 (1.22–1.86)		1.16 (0.93–1.46)	
Number of initial recurrence sites				
Solitary	1	<0.001	1	<0.001
Multiple	1.97 (1.63–2.38)		1.94 (1.56–2.41)	
Breakdown of treatment for initial recurrence				
Radical local treatment	1		1	
Nonradical local treatment	1.40 (1.17–1.68)	<0.001	1.32 (1.07–1.61)	0.008

^†^ Cox regression models for comparison of post‐recurrence cure among groups.

CI, confidence interval; HR, hazard ratio.

A total of 97 patients achieved five‐year survival after recurrence. Clinicopathological characteristics of the five‐year survivors and those who were not alive after five‐years are shown in Table 1. Compared to the nonsurvivors, the five‐year survivors had a significantly higher prevalence of solitary recurrence and radical local treatment for initial recurrent lesions.

### Associated factors for post‐recurrence cure

A total of 97 patients achieved five‐year survival after recurrence, and 24 patients (4.5%) were cured after recurrence. The median post‐recurrence follow‐up duration for post‐recurrence cure was 151 (range 85–275) months. The five‐year post‐recurrence survivors were classified as those with post‐recurrence cure (24 patients) and those with non‐post‐recurrence cure (73 patients); these patients were compared with PRS curves.

**Figure 3:**
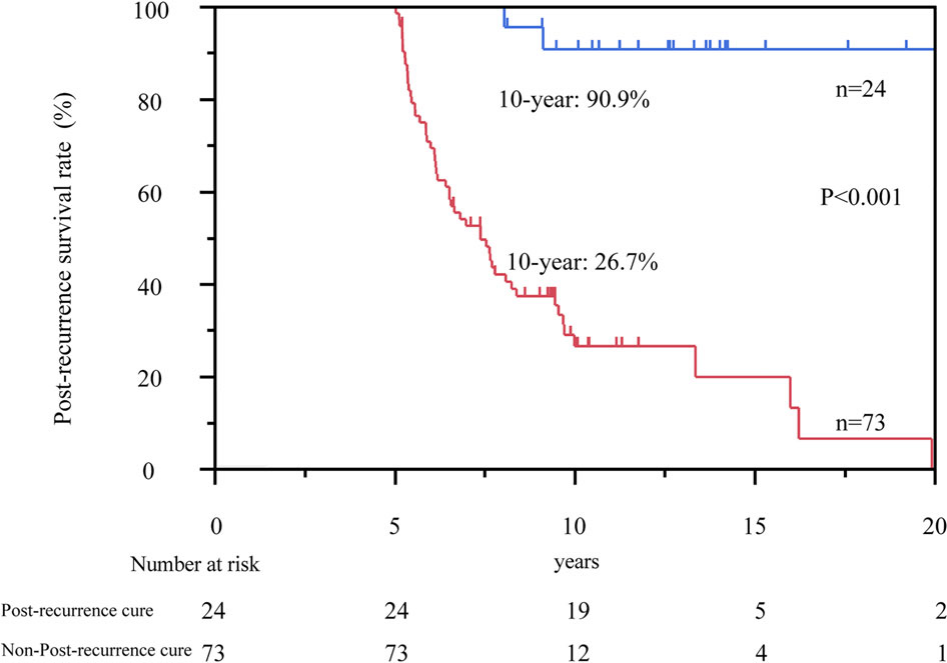
Kaplan–Meier curves for post‐recurrence survival (PRS) in patients with and without post‐recurrence cure who achieved five‐year survival after recurrence. The 10‐year PRS rate in patients with post‐recurrence cure was 90.9%. The 10‐year PRS rate was 26.7% in patients who failed to achieve post‐recurrence cure. 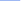
post‐recurrence cure and 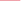
non‐post‐recurrence cure.

Post‐recurrence‐cure patients had significantly better 10‐year PRS than the non‐post‐recurrence‐cure patients (90.9% vs. 26.7%; *P* < 0.001, Fig 3).

A comparison of patients with and without post‐recurrence cure is shown in Table 3. The solitary recurrent lesions and local treatment at the initial recurrence site were significantly higher in those with post‐recurrence cure.

**Table 3:** Comparison of patients with and without post‐recurrence cure

	Post‐recurrence cure	Non‐post‐recurrence cure	
Characteristics	(*n* = 24)	(*n* = 73)	*P*‐value
Age at first surgery, years, median, range	59 (26–77)	64 (27–83)	0.273^†^
Age at recurrence, years, median, range	62 (28–77)	66 (28–86)	0.167^†^
Sex			
Male	14 (58.3%)	37 (50.7%)	0.515
Female	10 (41.7%)	36 (49.3%)	
Smoking history			
Yes	14 (58.3%)	36 (49.3%)	0.443
No	10 (41.7%)	37 (50.7%)	
Histology			
Adenocarcinoma	22 (91.7%)	63 (86.3%)	0.489
Others	2 (8.3%)	10 (13.7%)	
Pathological stage			
I	9 (37.5%)	25 (34.3%)	0.084
II–III	15 (62.5%)	48 (65.8%)	
Adjuvant chemotherapy			
Yes	6 (25.0%)	14 (19.2%)	0.374
No	18 (75.0%)	59 (80.8%)	
Recurrence‐free survival, months, median, range	23 (2–69)	24 (2–213)	0.217^†^
Initial recurrence site			
Locoregional recurrence	5 (20.8%)	30 (41.1%)	0.073
Systemic recurrence	19 (79.2%)	43 (58.9%)	
Number of initial recurrence sites			
Multiple	3 (12.5%)	36 (49.3%)	0.001
Solitary	21 (87.5%)	37 (50.7%)	
Breakdown of treatment for initial recurrence			
Radical local treatment	24 (100%)	48 (65.8%)	0.001
Nonradical local treatment	0	25 (34.3%)	

^†^ Student's *t*‐test.

In patients with post‐recurrence cure, solitary recurrent lesions were present in 21 patients (87.5%), while two recurrent lesions were present in the other three patients (12.5%). All patients with post‐recurrence cure received only radical local treatment, and systemic chemotherapy including molecular targeted therapy was not given after recurrence.

### Lesion characteristics and treatment of recurrence in patients with post‐recurrence cure

The recurrence site and treatment of all recurrence patients and post‐recurrence cure patients is shown in Table 4. The recurrence sites of post‐recurrence cure patients were lymph node, lung, brain, and bone. Regarding post‐recurrence cure rates by organ, both systemic recurrence and locoregional recurrence were higher in lymph node recurrence patients (systemic recurrence: 15.4% [2/13], locoregional recurrence: 8.6% [3/35]).

**Table 4:** Sites of recurrence and treatment of recurrence patients and post‐recurrence cure patients

		Treatment					
		Radical local treatment					
Initial recurrence site	Number of patients	Resection	Resection with radiation therapy	Radiation therapy	Others	Chemotherapy	Best supportive care
Systemic recurrence	407 (19)^†^	37 (6)	13 (2)	187 (11)	2	91	77
Single organ	241 (18)	31 (6)	7 (2)	113 (10)	2	47	41
Adrenal grand	6			2		4	
Bone	43 (1)	2	1	33 (1)		4	3
Brain	65 (8)	5 (1)	3 (1)	56 (6)			1
Liver	6	3				1	2
Lung	92 (7)	17 (5)		11 (2)	2	33	29
Lymph node (Extrathoracic)	13 (2)		1 (1)	8 (1)		3	1
Others	16	4	2	3		2	5
Multiple organs	166 (1)	6	6	74 (1)		44	36
Locoregional recurrence	128 (5)	14 (1)	2	58 (4)	2	29	23
Single organ	112	13	2	51	2	24	20
Lung	49 (2)	12 (1)	1	13 (1)	2	13	8
Lymph node	35 (3)		1	22 (3)		7	5
Pleura	19	1		9		2	7
Trachea	9			7		2	
Multiple organs	16	1		7		5	3

^†^ Numbers in parentheses are post‐recurrence cure patients.

There was one patient with recurrence in two lymph node sites including the mediastinal lymph nodes and contralateral supraclavicular lymph nodes. The lung recurrence involved the ipsilateral, contralateral, and bilateral lungs in two, six, and one patient, respectively. Only one patient had recurrence in two locations in the brain, whereas the remaining patients with brain recurrences had solitary brain lesions. Surgical resection was performed in nine patients with post‐recurrence cure.

## Discussion

In this study, patients who sustained cancer‐free status for five years after treatment for recurrent lesions and did not develop second recurrence were considered to be cured after recurrence. All post‐recurrence cure patients received only radical local treatment, and the solitary recurrent lesions and local treatment at the initial recurrent sites were significantly associated with post‐recurrence cure.

The standard treatment for recurrence after curative resection of NSCLC is systemic chemotherapy; however, long‐term survival after recurrence remains poor.^4^, ^5^, ^6^ The long‐term survival after recurrence was also poor in this study. Several recent studies have revealed that the introduction of molecular targeted agents or immune checkpoint inhibitors as systemic therapy as well as chemotherapy has led to significant improvements in survival after recurrence.^17^, ^18^, ^19^, ^20^, ^21^, ^22^, ^23^, ^24^, ^25^ Additionally, salvage SABR for isolated lung recurrence of NSCLC has been shown to be effective and comparable to the historically reported outcomes of patients with early stage primary NSCLC treated with definitive SABR.^26^

Furthermore, the recently proposed concept of oligo‐recurrence suggests that patients diagnosed with cancer and one to five metastatic or recurrent lesions which can be treated by local therapy may achieve long‐term survival or cure.^27^, ^28^ A comparison of patients with oligo‐recurrence to those with multiple recurrences has revealed that the PRS was better in oligo‐recurrence cases compared to those with multiple recurrences and was not dependent on the site of recurrence.^11^ This observation raises the possibility that survival may be prolonged by proactive local treatment of single or a few distant metastases such as those in the lung, brain, and adrenal glands in patients with controlled primary lesions.^8^, ^9^, ^10^, ^11^, ^12^, ^13^ Thus, recent progress in treatment approaches has led to improvements in the prognosis of recurrent NSCLC.

However, no studies to date have elucidated the characteristics of patients with NSCLC who have achieved post‐recurrence cure after the treatment for recurrence. How to consider the post‐recurrence patient after treatment for recurrent lesions as cured is an important issue. The post‐recurrence cure patients did not recognize any second recurrence after treatment for recurrent lesions even long‐term observation in the study. Thus, these cases are considered to have been cured after recurrence.

Multiple recurrences were observed in three patients with post‐recurrence cure, all of whom had two recurrent lesions, and three or more recurrent lesions were not observed in any of the patients. These multiple recurrences included two lung lesions in one patient, two brain lesions in one patient, and two regional lymph node lesions in one patient, reflecting that the recurrences were limited to one organ, even in patients with multiple recurrences.

Optimal treatment options for postoperative lung cancer recurrence are not sufficiently evidence‐based.^29^ Interestingly, post‐recurrence cure was not dependent on the stage of the primary lung cancer and site of recurrence, but depended on the number of recurrent lesions and the local treatment used for recurrent lesions. In this study, patients with a solitary or few recurrence sites of NSCLC achieved post‐recurrence cure with only radical local treatment of surgery or radiation therapy. Therefore, even radical local treatment alone can achieve a cure after recurrence. In patients with a solitary or a few recurrence sites cases, radical local treatment can be expected improve the prognosis and even achieve post‐recurrence cure.

Although oligo‐recurrence was observed, it is considered that the cancer cells are not disseminated throughout the body and are present in few organs. In post‐recurrence cure cases, systemic therapy was not performed for recurrent lesions, and cure was achieved only with radical local treatment, suggesting that the cancer cells were limited to recurrent lesions. This result supported the concept of oligo‐recurrence.

There are several limitations in the present study. First, this was a single‐institute, retrospective study, and the imaging modalities were different across the study cohort, although all patients underwent systemic examination to confirm recurrence. Second, the choice of treatment after recurrence was influenced by many factors such as the recurrence location as well as the patient's performance status, social background, and preference. Therefore, other factors may have affected the response to treatment management of recurrence. Third, the number of patients included was relatively small, which may have contributed to the lower statistical power. Fourth, incidence of patients with post‐recurrence cure is rare and this study included older patients.

In conclusion, in this cohort of 535 patients with NSCLC, post‐recurrence cure was observed in 4.5% of those who developed recurrence. The recurrent lesions were mostly solitary, and all patients with post‐recurrence cure received only radical local treatment for recurrent lesions. Radical local treatment can be expected to achieve cure in some patients with solitary recurrent of NSCLC that should be confirmed in large prospective trials.

## Disclosure

This research did not receive any specific grant from funding agencies in the public, commercial, or not‐for‐profit sectors.
